# Multistep flow synthesis of vinyl azides and their use in the copper-catalyzed Huisgen-type cycloaddition under inductive-heating conditions

**DOI:** 10.3762/bjoc.7.168

**Published:** 2011-10-20

**Authors:** Lukas Kupracz, Jan Hartwig, Jens Wegner, Sascha Ceylan, Andreas Kirschning

**Affiliations:** 1 Institute of Organic Chemistry, Leibniz University Hannover, Schneiderberg 1b, 30167 Hannover, Germany

**Keywords:** flow reactor, inductive heating, iodine azide, polymer-supported reagents, vinyl azides

## Abstract

The multistep flow synthesis of vinyl azides and their application in the synthesis of vinyltriazoles is reported. The synthesis relies on a stable polymer-bound equivalent of iodine azide that serves to carry out 1,2-functionalization of alkenes in a telescope flow protocol. The intermediate 2-iodo azides are subjected to a DBU-mediated polymer-supported elimination step yielding vinyl azides in good yield. The third step involves the formation of vinyl triazoles by a copper-catalyzed Huisgen-"click" cycloaddition. The required heat is generated by electromagnetic induction based on copper. Copper serves both as heatable as well as catalytically active packed-bed material inside the flow reactor.

## Introduction

**Scheme 1:**
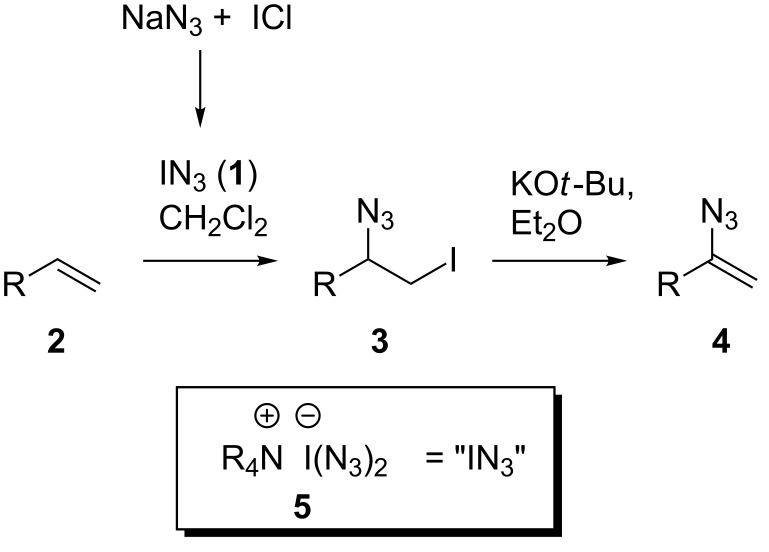
Hassner's synthesis of vinyl azides and a stable, nonexplosive analogue **5** of iodine azide (**1**).

Azides are highly versatile organic functional groups and their preparation and their reactivity are well explored [1]. In contrast, the synthesis of vinyl azides is far away from being well established despite the fact that an even richer chemistry than that which has lately been developed for azides can be envisaged as vinyl azides bear the additional alkene moiety. One of the first synthetic studies on vinyl azides was disclosed by L'Abbé as early as 1975 [1]. Surprisingly, only a very few applications based on these potentially very useful functional groups have been reported to date. Lately, Yu et al. [2] disclosed the synthesis of pyrazoles, while Chiba et al. employed vinyl azides for the Mn(III)-mediated synthesis of different azaheterocycles [3–4]. Furthermore, vinyl azides can be converted into the corresponding 2*H*-azirines by thermolysis or alternatively by photolysis [5]. The highly reactive azirines can further react as dipolarophiles, dienophiles, electrophiles or nucleophiles [6] thereby accessing oxazoles and isoxazoles [7]. In addition, 2*H*-azirines were also used in the Hemetsberger reaction, which yields indoles [8]. However, straightforward and safe methods for the preparation of vinyl azides are still scarce. In fact, in most cases the synthesis involves the generation of toxic and explosive azido intermediates. The most frequently used batch process for the generation of vinyl azides **4** is the two-step protocol developed by Hassner et al. [9] through the in situ reaction of sodium azide with iodine chloride in dichloromethane or another polar solvent (Scheme 1). Thus, it includes the generation of hazardous and highly explosive iodine azide (IN_3_, **1**).

**Scheme 2:**
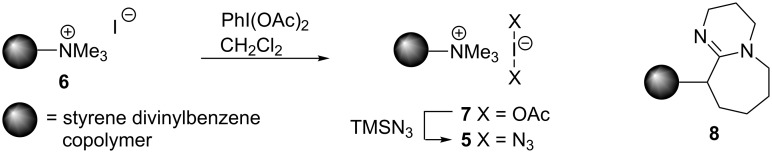
Preparation of polymer-bound bisazido iodate(I) **5** and polymer-bound 1,8-diaza-[5.4.0]bicyclo-7-undecene **8**.

In their synthesis of carbamates Wirth and coworkers [10] recently showed that some of the practical problems associated with iodine azide can be circumvented or minimized by generating and immediately consuming iodine azide under flow conditions. Because of low yields, this ingenious process requires further optimization. This and other examples [11–13] principally demonstrate that flow chemistry is an ideal enabling technology [14] for generating and utilizing hazardous azido reagents because only small amounts are generated at a time and are subsequently consumed in situ. The other benefits of flow chemistry, when working with highly reactive reagents, are the better heat-transfer characteristics due to a larger surface-to-volume ratio, as well as the increased mixing efficiency [15–24]. To practically eliminate the generation of explosive iodine azide we developed the iodine azide transfer reagent **5** based on ammonium iodate(I) complexes [25]. As depicted in Scheme 2 the reagent can be prepared in three steps without the generation of free iodine azide (**1**). Importantly, this reagent can easily be prepared as an ion-exchange resin based on Amberlyst A-26 [26]. Chemically, this reagent behaves like iodine azide (**1**), but in contrast it is not explosive and is storable for weeks without a substantial loss of activity [27].

In this report, we disclose the first two-step flow synthesis of vinyl azides based on functionalized polymers **5** and **8**. The protocol starts from alkenes, which are transformed by a 1,2-addition of iodine azide and then to the corresponding vinyl azides. Furthermore, for the first time we present the copper-mediated Huisgen-type “click” cycloaddition of vinyl azides with alkynes to yield vinyl triazoles under inductive-heating conditions.

## Results and Discussion

### Synthesis of vinyl azides

Our study commenced with the preparation and utilization of polymer-bound iodate(I) complex **5** as packed-bed material in a flow device. Here, the polymer served as an electrophilic reagent for the mild 1,2-azidoiodination of alkenes under flow conditions. A solution of the alkene dissolved in dichloromethane (0.2 M) was passed, at room temperature, through a glass reactor (12 cm length and 8.5 mm internal diameter) filled with polymer **5** (5 g; theoretical loading = 3.5 mmol/g) that had been prepared as reported before [26]. Reaction conditions listed in Table 1 are optimized with respect to loading, flow rates and temperature. Higher temperatures and solvents other than dichloromethane were not beneficial due to degradation of the functionalized polymer **5** or the generation of byproducts. The resulting β-iodo azides **3** were isolated in moderate to very good yields. In two cases, the flow procedure was compared with the corresponding batch experiment. Yields were comparable but reaction times were shorter for the flow process. The dead volume of the polymer-filled reactor is about 4.5 mL and thus the theoretical residence times in the reactor at the given flow rates are between 1.5 h to 3.5 h.

**Table 1:** Azido iodination of alkenes **2a**–**f** under flow conditions with polymer-bound bisazido iodate(I) complex **5**.

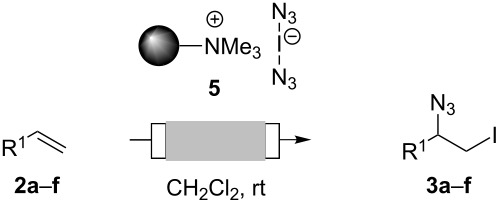						

entry	substrate	flow rate [mL/min]	equiv **5**^a^	product (racemic)	yield flow^b^	*t*, (yield) batch^b^

1	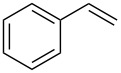 **2a**	0.05	5	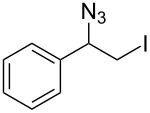 **3a**	98%	15 h (97%)
2	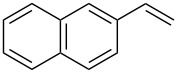 **2b**	0.04	5	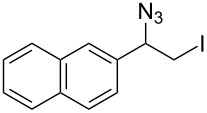 **3b**	91%	20 h (95%)
3	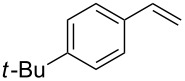 **2c**	0.03	5	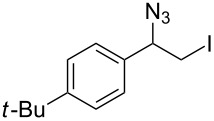 **3c**	61%	
4	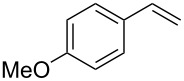 **2d**	0.02	5	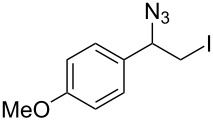 **3d**	75%	
5	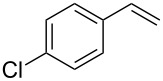 **2e**	0.02	6	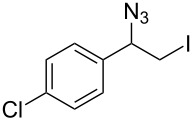 **3e**	78%^c^	
6	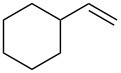 **2f**	0.02	7	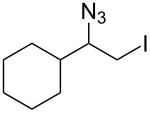 **3f**	70%^d^	

^a^Equiv of **5** refers to the theoretical loading based on polymer-bound ammonium groups of Amberlyst A-26; ^b^Isolated yields of pure products after evaporation of the solvent and in some cases followed by chromatographic purification; ^c^Contains about 12% starting material; ^d^Contains 15% of bisazido product.

Mechanistically the good regioselectivity is based on the generation of the more stable carbenium ion after electrophilic attack of the iodonium species on the olefinic double bond. In cases of aliphatic alkenes without aryl substituents, prolonged reaction times were observed, which could be shortened by use of more equivalents of the functionalized polymer **5**. However, when a larger excess of reagent **5** was applied we observed the formation of the diazido byproducts, which are likely formed after a second nucleophilic-substitution step of the azide anion onto the intermediate iodo azide.

Next, we studied the elimination step that should yield the target vinyl azides. Hassner et al. [9] relied on potassium *tert-*butoxide as a base, which indeed worked well in diethyl ether as the solvent in our initial batch experiments (Table 2, entry 1). However, as the first flow step was performed in dichloromethane, we tested this solvent for the elimination in order to achieve a telescope process. Unfortunately, we encountered decomposition of the starting material (Table 2, entry 2) so that, again, an optimized protocol had to be found.

**Table 2:** Optimization of the elimination protocol and formation of vinyl azide **4a** under batch conditions (entries 1–7) and as a flow protocol (entry 8).

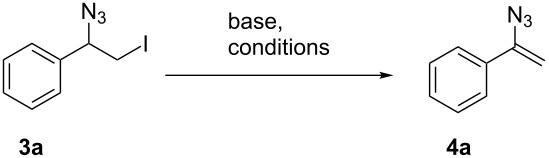					

entry	base	solvent	*T* [°C]	*t* [h]	yield^a^

1	1.5 equiv KO*t*-Bu	Et_2_O	rt	2	95%
2	1.5 equiv KO*t*-Bu	CH_2_Cl_2_	rt	2	decomposition
3	5 equiv K_2_CO_3_	CH_2_Cl_2_	rt	18	5%
4^b^	5 equiv K_2_CO_3_	CH_2_Cl_2_	60	18	23%
5	2.5 equiv DIPEA^c^	DMF	60	2	92%
6	2 equiv DBU	CH_2_Cl_2_	rt	1.5	92%
7	2 equiv PS–DBU^d^	CH_2_Cl_2_	rt	1.5	93%
8^e^	2 equiv PS–DBU	CH_2_Cl_2_	rt	0.04 mL/min	complete transformation

^a^Isolated yields; ^b^Reaction was carried out in a microwave-compatible tube heated in an oil bath, ^c^DIPEA = diisopropylethyl amine; ^d^PS–DBU = polystyrene-bound 1,8-diaza-[5.4.0]bicyclo-7-undecene (**8**); ^e^Flow process: Glass reactor (12 cm length and 8.5 mm internal diameter) filled with polymer **8** (0.5 g; theoretical loading = 1.15 mmol/g).

The best results in dichloromethane were achieved when 1,8-diazabicyclo[5.4.0]undec-7-ene (DBU, **8**) was employed as a base (Table 2, entry 6). The polymer-bound variant of DBU (PS–DBU) also gave excellent results (Table 2, entry 7) such that these conditions could directly be used for the flow process (Table 2, entry 8).

**Scheme 3:**
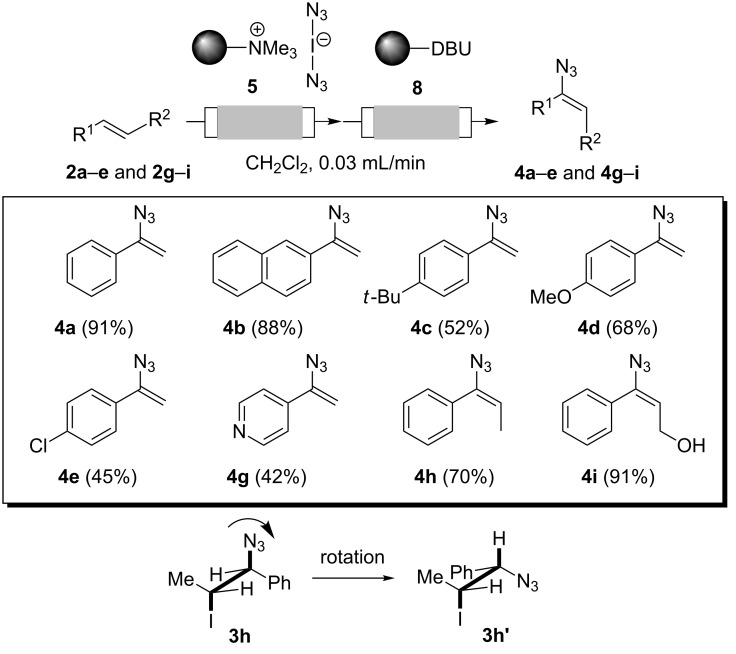
Two-step protocol for the preparation of vinyl azides **4a**–**e** and **4g**–**i** under flow conditions.

Finally, both flow reactors were telescoped, which allowed us to prepare vinyl azides **4a–e** and **4g**–**i** in one flow process starting from alkenes **2a**–**e** and **2g**–**i** (Scheme 3). Advantageously, isolation and purification of intermediate iodo azides **3** was avoided. As a consequence we achieved improved yields compared to those obtained for individual steps. When starting from (*E*)-configured alkenes **2h** and **2i**, configurationally pure vinyl azides **4h** and **4i** with *syn*-orientation for both alkyl substituents were formed, as judged by nuclear Overhauser effect (NOE) experiments (Supporting Information File 1). The stereochemical outcome of this addition–elimination process may be rationalized by assuming an *anti*-addition of iodine azide onto the π-bond. After rotation along the C–C σ-bond (**3h** to **3h'**) *anti*-orientation of the proton and iodine allows for facile base-mediated elimination. This results in trisubstituted alkenes **4h** and **4i** owing to the observed stereochemistry.

**Scheme 4:**
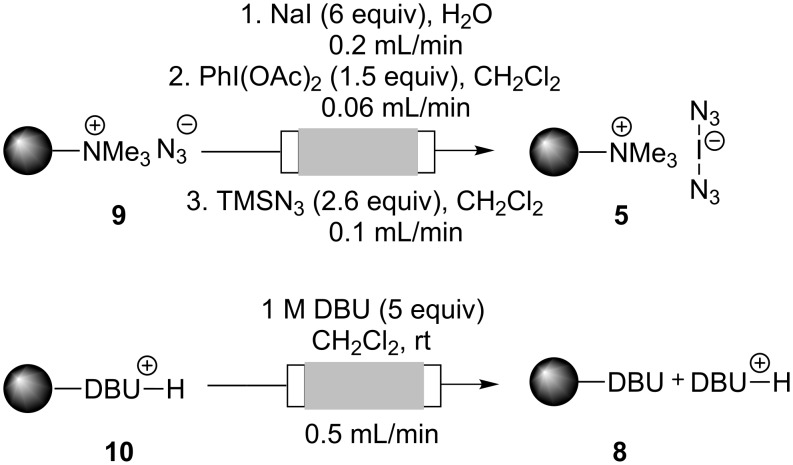
Regeneration of functionalized polymers **5** and **8**.

Both of the functionalized polymers **5** and **8** are ideally suited for regeneration of the active species by employing simple flushing protocols without having to change the principal setup. Thus, azide-loaded ion-exchange resin **9**, which is supposed to be the main species after azido iodination, was regenerated by first ion exchange to the iodide form **2**. Next, oxidation to the bisacetoxy iodate(I)-complex **7** was achieved by treatment with diacetoxyiodo benzene. Finally, pumping of a solution of trimethylsilyl azide (TMSN_3_) in dichloromethane afforded the functionalized polymer **5**, which could be used for 1,2-functionalization of alkenes with the same efficiency as described in Table 1. In addition the protonated form of PS–DBU **10** was regenerated to PS–DBU **8** by rinsing the reactor with a 1 M solution of DBU (Scheme 4).

### Copper-catalyzed Huisgen-type cycloadditions

The copper-catalyzed Huisgen-type cycloaddition (CuAAC) is a general and useful method for the synthesis of 1,4-disubstituted-1,2,3-triazoles and is based on the 1,3-dipolar cycloaddition of alkynes and azides [28]. Besides Cu(I) sources also Cu(0) sources, such as copper wire [29] or copper-on-charcoal (Cu/C) [30], can serve as a catalytic source that promotes the CuAAC. Bogdan et al. combined this observation with flow technology by using a custom-made heated copper flow reactor [31]. We successfully implemented the CuAAC by inductively heating copper wire inside a flow microreactor [32]. A key benefit of this technology is that the copper metal is directly and instantaneously heated inside the reactor, which results in a higher reactivity than with conventionally heated elemental copper [32]. These results prompted us to investigate the reaction of vinyl azides **4** in the copper-catalyzed Huisgen-type cycloaddition. Importantly, this cycloaddition has not been reported for vinyl azides so far.

Therefore, a series of experiments had to be conducted in order to find the best conditions for achieving triazole formation. The temperature was measured on the reactor surface by means of an IR pyrometer. We chose 2-(1-azidovinyl)naphthalene (**4b**) and phenylacetylene (**11a**) as reaction partners for optimization. First, we had to find the best copper source (Table 3). We found that Cu-turnings gave complete conversion and a good isolated yield (Table 3, entry 2) for which the larger surface area can be held responsible. Flow rates of 0.05 mL/min or higher led to very low or zero conversion. Increasing the temperature led to decomposition of the reactants (Table 3, entries 4 and 5) as noted above. Employing DMF as the solvent gave the best results (Table 3, entries 6 and 7). Addition of a base or CuSO_4_ in order to create more of the active copper species did not turn out to be beneficial for this transformation (Table 3, entries 8 and 9).

**Table 3:** Optimization of the reaction of vinyl azide **4b** in the inductively heated (IH) copper-catalyzed Huisgen-type cycloaddition.

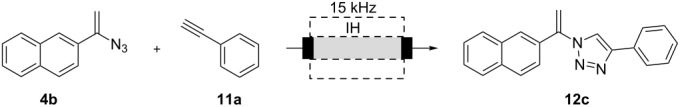					

entry	catalyst	solvent	flow rate [mL/min]	*T* [°C]	yield [%]

1	Cu-wire	DMF	0.04	70	32
**2**	**Cu-turnings**	**DMF**	**0.04**	**80**	**72**
3	Cu-turnings	DMF	0.07	80	44
4	Cu-turnings	DMF	0.04	100	23
5	Cu-turnings	DMF	0.04	110	10
6	Cu-turnings	acetone	0.04	80	59
7	Cu-turnings	dioxane	0.04	80	55
8	Cu-turnings/0.2 equiv CuSO_4_	DMF/H_2_O 1:1	0.04	80	58
9	Cu-turnings/1 equiv DIPEA	DMF	0.04	80	68

**Scheme 5:**
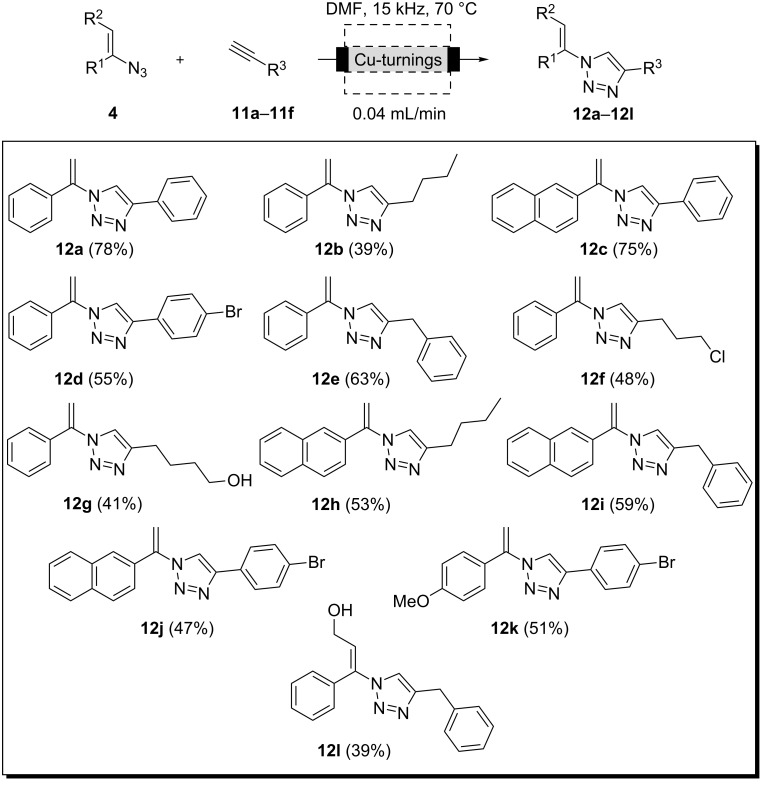
Preparation of triazoles **12a**–**l** by using inductively heated copper turnings as a packed-bed material inside flow reactors.

However, with the optimized flow protocol in hand, alkyl and aryl substituted triazoles **12 a**–**l** were prepared (Scheme 5)

It can be envisaged that this three step sequence can be alternatively arranged in a different order. Thus, after the azido iodination, first the cycloaddition is conducted, followed by the elimination step. In fact, we tested this route but experienced substantial difficulties. First, we were unable to achieve the cycloaddition in dichloromethane, and second, this transformation only proceeded in poor yields.

## Conclusion

In summary, we developed the first telescope protocol for preparing vinyl azides starting from the corresponding alkenes, which was conducted in the flow mode. This two-step protocol was achieved by employing two functionalized polymers that served as a packed-bed material inside the reactor. Additionally, we showed that copper-catalyzed vinyl triazole formation of these vinyl azides in the presence of alkynes is possible by using elemental copper as an inductively heatable material. The method avoids the use of explosive and hazardous iodine azide.

## Experimental

### 

#### General procedure for azido iodination of alkenes **2** under flow conditions

A glass reactor (12 cm length and 8.5 mm internal diameter) was filled with polymer-bound iodate(I) complex **5** (5 g; theoretical loading = 3.5 mmol/g) and protected from light with aluminum foil. The reactor (void volume: 4.5 mL) was connected to the pump and, at the outlet side, to a collection flask. The system was first flushed with dry CH_2_Cl_2_ (8 mL, 0.5 mL/min). Then, a solution of styrene (**2a**) (364 mg, 3.5 mmol) in dry CH_2_Cl_2_ (17.5 mL) was pumped through the reactor at a flow rate of 0.04 mL/min. Then, 10 mL of CH_2_Cl_2_ was used to wash the reactor (5 mL, 0.04 mL/min + 5 mL, 0.5 mL/min). Product **3a** (936 mg, 3.4 mmol, 98%) was directly obtained after evaporation of the combined organic phases.

#### General procedure for the two-step preparation of vinyl azides **4** under flow conditions

A glass reactor (12 cm length and 8.5 mm internal diameter) filled with polymer-bound iodate(I) complex **5** (5 g; theoretical loading = 3.5 mmol/g) and a second identical flow reactor, which was filled with a slurry of polystyrene-bound 1,8-diaza-[5.4.0]bicyclo-7-undecene (**8**) (4 g; theoretical loading = 1.15 mmol/g) in dry CH_2_Cl_2_ (5 mL), were telescoped and protected from light with aluminum foil. The system was connected to the pump and, at the outlet side, to a collection flask. After priming with CH_2_Cl_2_ (10 ml), a solution of styrene (**2a**) (364 mg, 3.5 mmol) in dry CH_2_Cl_2_ (17.5 mL) was flushed at 0.04 mL/min. Then, 10 mL more of CH_2_Cl_2_ was pumped through the reactor (5 mL, 0.04 mL/min + 5 mL, 0.5 mL/min). The crude product **4a** (462 mg, 3.2 mmol, 91%) was isolated after evaporation of the solvent. If necessary (see Supporting Information File 1), the crude product was purified by column chromatography (petroleum ether/ethyl acetate).

#### Regeneration of functionalized polymer-bound iodate(I) complex **5** under flow conditions

The reactor with azide-loaded ion-exchange resin **9** (5 g; theoretical loading = 3.5 mmol/g) was connected to the pump and, at the outlet side, to a collection flask. The system was first flushed with water (10 mL, 0.5 mL/min). A solution of NaI (15.7 g, 105 mmol) in water (20 mL) was pumped through the reactor at flow rate of 0.15 mL/min. Then the reactor was successfully washed with 10mL each of water, acetone and finally with CH_2_Cl_2_ at a flow rate of 0.5 mL/min.

In the second step the system was flushed with a solution of PhI(OAc)_2_ (8.5 g, 26.3 mmol) in 60 mL of dry CH_2_Cl_2_ at a flow rate of 0.06 mL/min. Then, the reactor was washed with 20 mL of CH_2_Cl_2_ at a flow rate of 0.5 mL/min.

In the third step, a solution of TMSN_3_ (6.0 mL, 45.5 mmol) in 20 mL of dry CH_2_Cl_2_ was pumped through the reactor at a flow rate of 0.10 mL/min. Finally, the reactor was washed with 20 mL of CH_2_Cl_2_ at a flow rate of 0.5 mL/min.

#### General procedure for the copper-catalyzed Huisgen-type cycloaddition under flow conditions

A glass reactor (12 cm length and 8.5 mm internal diameter) packed with copper turnings (12 g) was encased within the inductor. The reactor was connected to the pump and, at the outlet side, to a collection flask. The system was flushed with DMF (flow rate 0.04 mL/min), and the temperature was adjusted to 70 °C regulating the PMW (pulse-width modulation). A sample loop was filled with a solution of vinyl azides **4** (0.3 mmol, 1 equiv) and alkynes **11** (0.45 mmol, 1.5 equiv) in DMF (0.5 mL). After the flow and temperature values reached a steady state, the solution was pumped through the system. Washing was continued until no product was detected at the outlet as judged by TLC. The resulting solution was diluted with 50 mL of ethyl acetate and washed with water and brine (3 × 60 mL). The organic layers were dried over anhydrous MgSO_4_ and evaporated under vacuum. The crude product was then purified by flash chromatography (petroleum ether/ethyl acetate) to yield the pure products **12**.

## Supporting Information

The Supporting Information provides details on individual reactions and analytical data.

File 1 Details on individual reactions and analytical data.
