# Supplementary material for: Multistep flow synthesis of vinyl azides and their use in the copper-catalyzed Huisgen-type cycloaddition under inductive-heating conditions
Source: Beilstein J Org Chem. 2011 Oct 20;7:1441–8. doi: 10.3762/bjoc.7.168 (PMC3205773; doi:10.3762/bjoc.7.168)
Supplement: File 1 — Details on individual reactions and analytical data. [file Beilstein_J_Org_Chem-07-1441-s001.pdf]

Supporting Information

for

Multistep flow synthesis of vinyl azides and their use in the copper-catalyzed Huisgen-type cycloaddition under inductive-heating conditions

Lukas Kupracz, Jan Hartwig, Jens Wegner, Sascha Ceylan, Andreas Kirschning*

Address: Institute of Organic Chemistry, Leibniz University Hannover, Schneiderberg 1b, 30167 Hannover, Germany

Email: Lukas Kupracz - lukas.kupracz@oci.uni-hannover.de; Jan Hartwig - jan.hartwig@oci.uni-hannover.de; Jens Wegner - jens.wegner@oci.uni-hannover.de; Andreas Kirschning* - andreas.kirschning@oci.uni-hannover.de

* Corresponding author

Details on individual reactions and analytical data

Experimental

NMR spectra were recorded on a Bruker AV400 spectrometer at 400 MHz (^1H NMR) and at 100 MHz (^{13}C NMR) in $CDCl_3$. Spectra are reported as values in ppm relative to (residual undeuterated) solvent signal as internal standard. Mass spectra (EI) were obtained at 70 eV with a type Finnigan Mat 312 or (ESI) with a type Q-Tof Premier (Waters). Melting points were determined in open glass capillaries with an OptiMelt from Stanford Research Systems (Sunnyvale, USA) and are uncorrected. Analytical thin-layer chromatography was performed with precoated silica gel 60 F_{254} plates (Merck, Darmstadt), and the spots were visualized with UV light at 254 nm or by staining with H_2SO_4/4-methoxybenzaldehyde in ethanol. Flash column chromatography was performed on a Biotage System.

The inductors were designed and manufactured by IFF GmbH (Ismaning, Germany). Pumps were obtained from Knauer GmbH (Berlin, Germany). The temperature was measured on the reactor surface by means of an IR pyrometer obtained from optris GmbH (LaserSight model). Copper turnings were purchased from Sigma-Aldrich. Commercially available reagents and dry solvents (DMF, CH_2Cl_2) were used as received.

Analytical data

(1-Azido-2-iodoethyl)benzene (3a)

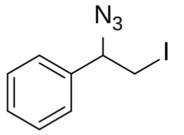

Compound **3a** was prepared in 98% yield as a colorless oil; ^1H NMR (400 MHz, $CDCl_3$) δ 7.47–7.29 (m, 5H), 4.72 (t, *J* = 7.0 Hz, 1H), 3.40 (d, *J* = 7.0 Hz, 2H); ^{13}C NMR (100 MHz, $CDCl_3$) δ 137.8, 129.1, 126.6, 67.1, 8.0.

All data were in accordance with published data [1].

2-(1-Azido-2-iodoethyl)naphthalene (3b)

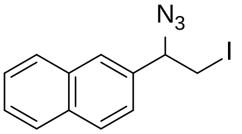

Compound **3b** was prepared in 91% yield as a pale yellow oil; ^{1}H NMR (400 MHz, $CDCl_3$) δ 7.92–7.87 (m,3H), 7.81 (s, 1H), 7.57–7.54 (m, 2H), 7.42 (dd, *J* = 8.2, 1.4 Hz, 2H), 4.90 (t, *J* = 6.8 Hz, 1H), 3.49 (d, *J* = 7.5 Hz, 2H); ^{13}C NMR (100 MHz, $CDCl_3$) δ 135.1, 133.4, 133.0, 129.1, 128.1, 127.8, 126.7, 126.3, 123.6, 67.3, 8.0.

All data were in accordance with published data [2].

1-(1-Azido-2-iodoethyl)-4-(*tert*-butyl)benzene (3c)

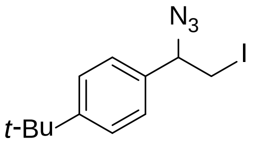

Compound **3c** was prepared in 61% yield as a colorless oil; ^{1}H NMR (400 MHz, $CDCl_3$) δ 7.07 (d, *J* = 8.2 Hz, 2H), 6.90 (d, *J* = 8.2 Hz, 2H), 4.35 (t, *J* = 7.5 Hz, 1H), 3.04–3.02 (m, 2H), 0.98 (s, 9H); ^{13}C NMR (100 MHz, $CDCl_3$) δ 152.0, 134.9, 126.2, 125.9, 67.0, 34.6, 31.2, 8.4; LRMS (EI) [M + H^+] calcd for $C_{12}H_{17}IN_3^+$, 330.0467; found, 330.

1-(1-Azido-2-iodoethyl)-4-methoxybenzene (3d)

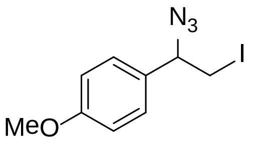

Compound **3d** was prepared in 75% yield as a colorless oil; ^{1}H NMR (400 MHz, $CDCl_3$) δ 7.28 (d, *J* = 8.5 Hz, 2H), 6.96 (d, *J* = 8.5 Hz, 2H), 4.65 (dd, *J* = 8.2, 5.1 Hz, 1H), 3.84 (s, 3H), 3.51 (dd, *J* = 12.6, 8.5 Hz, 1H), 3.43 (dd, *J* = 12.9, 5.1 Hz, 1H); ^{13}C NMR (100 MHz, $CDCl_3$) δ 160.0, 129.8, 128.2, 114.4, 65.0, 55.2, 8.4.

All data were in accordance with published data [3].

1-(1-Azido-2-iodoethyl)-4-chlorobenzene (3e)

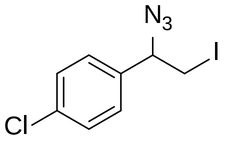

Compound **3e** was prepared in 78% yield as a colorless oil; ^{1}H NMR (400 MHz, $CDCl_3$) δ 7.41 (d, *J* = 8.2 Hz, 2H), 7.29 (d, *J* = 8.6 Hz, 1H), 4.72 (t, *J* = 6.8 Hz, 1H), 3.39 (d, *J* = 7.0 Hz, 2H); ^{13}C NMR (100 MHz, $CDCl_3$) δ 136.3, 134.8, 129.2, 128.0, 66.1, 7.8; LRMS (EI) [M + H^+] calcd for $C_8H_8ClN_3^+$, 307.9451; found, 308.

(1-Azido-2-iodoethyl)cyclohexane (3f)

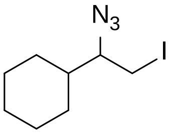

Compound **3f** was prepared in 70% yield as a colorless oil; ^{1}H NMR (400 MHz, $CDCl_3$) δ 3.77 (dd, *J* = 13.0, 6.5 Hz, 1H), 3.70 (dd, *J* = 13.0, 7.2 Hz, 1H), 3.38–3.32 (m, 1H), 3.28–3.24 (m, 1H), 1.82–1.58 (m, 5H), 1.36–1.09 (m, 5H); ^{13}C NMR (100 MHz, $CDCl_3$) δ 68.6, 42.4, 29.7, 28.0, 26.0, 7.1; LRMS (EI) [M + H^+] calcd for $C_8H_{15}IN_3^+$, 280.01293; found, 280.

(1-Azidovinyl)benzene (4a)

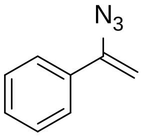

Compound **4a** was prepared in 91% yield as a yellow oil; ^{1}H NMR (400 MHz, $CDCl_3$) δ 7.58–7.56 (m, 2H), 7.36–7.33 (m, 3H), 5.44 (d, J = 2.0 Hz, 1H), 4.97 (d, *J* = 2.0 Hz, 1H); ^{13}C NMR (100 MHz, $CDCl_3$) δ 145.0, 134.2, 129.1, 128.4, 125.5, 98.0;

All data were in accordance with published data [4].

2-(1-Azidovinyl)naphthalene (4b)

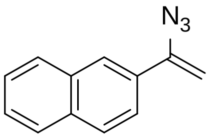

Compound **4b** was prepared in 88% yield as a colorless solid; mp 56 °C (Lit: 56–58 °C); ^{1}H NMR (400 MHz, $CDCl_3$) δ 8.05 (s, 1H), 7.80–7.92 (m, 3H), 7.67 (dd, *J* = 8.7, 1.9 Hz, 1H), 7.5 (d, *J* = 9.6 Hz, 1H), 7.50 (d, *J* = 2.4 Hz, 1H), 5.59 (d, *J* = 2.7 Hz, 1H), 5.07 (d, 2.4 Hz, 1H); ^{13}C NMR (100 MHz, $CDCl_3$) δ 145.0, 133.4, 133.0, 131.6, 128.7, 128.3, 127.7, 126.8, 126.6, 125.1, 123.2, 98.4; HRMS(ESI) [M + H^+] calcd for $C_{12}H_{10}N_3^+$, 196,0875; found, 196.0871.

All data were in accordance with published data [5].

1-(1-Azidovinyl)-4-(*tert*-butyl)benzene (4c)

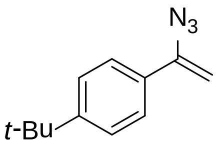

Compound **4c** was prepared in 52% yield as a colorless solid; mp 44 °C; ^{1}H NMR (400 MHz, $CDCl_3$) δ 7.49 (d, *J* = 8.5 Hz, 2H), 7.38 (d, *J* = 8.9 Hz, 2H), 5.4 (d, *J* = 2.4 Hz, 1H), 4.92 (d, *J* = 2.4 Hz, 1H), 1.32 (s, 9H); ^{13}C NMR (100 MHz, $CDCl_3$) δ 152.5, 145.1, 125.5, 125.4, 97.4, 34.8, 31.4; HRMS (ESI) [M + H^+] calcd for $C_{12}H_{16}N_3^+$, 202.1344; found, 202.1348.

1-(1-Azidovinyl)-4-methoxybenzene (4d)

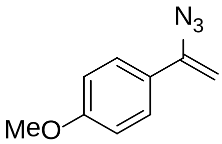

Compound **4d** was prepared in 68% yield as a colorless solid; mp 38 °C (Lit: 39–40 °C [6]); ^{1}H NMR (400 MHz, $CDCl_3$) *δ* 7,49 (d, *J* = 8.9 Hz, 2H), 6.87 (d, *J* = 8.9 Hz, 2H), 5.31 (d, *J* = 2.4 Hz, 1H), 4.86 (d, *J* = 2.4 Hz, 1H), 3.82 (s, 3H); ^{13}C NMR (100 MHz, $CDCl_3$) δ 160.5, 144.8, 127.1, 113.9, 96.3, 55.5 (one aromatic carbon is not resolved); HRMS (ESI) [M + H^+] calcd for $C_9H_{10}N_3O^+$, 176.0824; found, 176.0825.

All data were in accordance with published data [3].

1-(1-Azidovinyl)-4-chlorobenzene (4e)

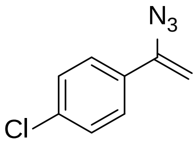

Compound **4e** was prepared in 45% yield as a colorless oil; ^{1}H NMR (400 MHz, $CDCl_3$) δ 7.44 (d, *J* = 8.7 Hz, 1H), 7.32 (d, *J* = 8.7 Hz, 1H), 5.43 (d, *J* = 2.6 Hz, 1H), 4.97 (d, *J* = 2.6 Hz, 1H); ^{13}C NMR (100 MHz, $CDCl_3$) δ 165.1, 140.2, 127.8, 113.9, 101.2, 98.1; HRMS (ESI) [M + H$^+$] calcd for $C_8H_7ClN_3^+$, 180.0328; found, 180.0331.

4-(1-Azidovinyl)pyridine (4g)

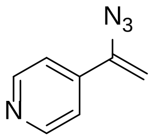

Compound **4g** was prepared in 42% yield as a yellow solid; mp 34 °C; ^{1}H NMR (400 MHz, $CDCl_3$,) δ 8.61 (d, *J* = 6.1 Hz, 2H), 7.44 (d, *J* = 6.1 Hz, 2H), 5.67 (d, *J* = 3.1 Hz, 1H), 5.13 (d, *J* = 3.1 Hz, 1H); ^{13}C NMR (100 MHz, $CDCl_3$) δ 154.1, 143.2, 141.6, 119.7, 100.9; HRMS (ESI) [M + H$^+$] calcd for $C_7H_7N_4^+$, 147.0671; found, 147.0670.

(*E*)-(1-Azidoprop-1-en-1-yl)benzene (4h)

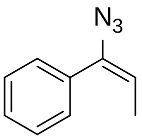

Compound **4h** was prepared in 70% yield as a colorless oil; ^{1}H NMR (400 MHz, $CDCl_3$) δ 7.30–7.44 (m, 5H), 5.48 (q, *J* = 7.3 Hz, 1H), 1.72 (d, *J* = 7.2 Hz, 3H); ^{13}C NMR (100 MHz, $CDCl_3$) δ 128.9, 128.7, 128.6, 112.2, 13.9; HRMS (ESI) [M + H$^+$] calcd for $C_9H_{10}N_3^+$,160.0875; found, 160.0874.

Table S1: NOE effects in **4h**.

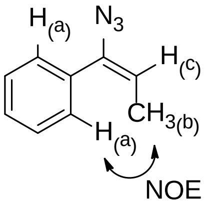

	rel. NOE effect [%]
H_b/H_c	1
H_b/H_a	0.79
H_c/H_b	–[a]
H_c/H_a	–[a]

[a]NOE not observed.

(*E*)-3-Azido-3-phenylprop-2-en-1-ol (4i)

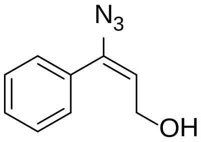

Compound **4i** was prepared in 91% yield as a colorless oil; ^{1}H NMR (400 MHz, $CDCl_3$) δ 7.39–7.45 (m, 3H), 7.31–7.35 (m, 2H), 5.63 (t, *J* = 7.5 Hz, 1H), 4.16 (d, *J* = 7.2 Hz, 2H); ^{13}C NMR (100 MHz, $CDCl_3$) δ 129.8, 129.5, 129.3, 128.7, 128.7, 115.2, 59.4; HRMS (ESI) [M + H^+] calcd for $C_9H_{10}N_3O^+$, 176.0824; found, 176.0821.

Table S2: NOE effects in **4i**.

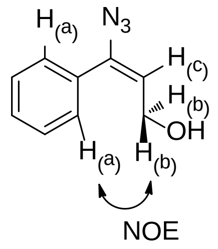

	rel. NOE effect [%]
H_b/H_c	1
H_b/H_a	0.98
H_c/H_b	0.42
H_c/H_a	–[a]

[a]NOE not observed.

4-Phenyl-1-(1-phenylvinyl)-1*H*-1,2,3-triazole (12a)

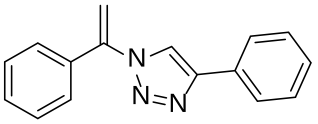

Compound **12a** was prepared in 78% yield as a yellow oil; ^{1}H NMR ($CDCl_3$) δ 7.85 (d, *J* = 7.5 Hz, 2H), 7.80 (s, 1H), 7.48–7.33 (m, 8H), 5.87 (d, J = 0.7 Hz, 1H), 5.56 (d, *J* = 0.7 Hz, 1H); ^{13}C NMR (100 MHz, $CDCl_3$) δ 147.6, 143.0, 134.6, 130.2, 129.9, 128.9, 128.3, 127.3, 125.8, 119.8, 109.4; HRMS (ESI) [M + H^+] calcd for $C_{16}H_{14}N_3$, 248.118; found, 248.120.

All data were in accordance with published data [7].

4-Butyl-1-(1-phenylvinyl)-1*H*-1,2,3-triazole (12b)

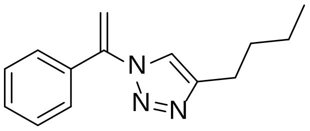

Compound **12b** was prepared in 39% yield as a yellow oil; ^{1}H NMR ($CDCl_3$) δ 7.43–7.37 (m, 3H), 7.33–7.30 (m, 2H), 7.31 (s, 1H), 5.76 (s, 1H), 5.48 (s, 1H), 2.75 (t, *J* = 7.6 Hz, 2H), 1.67 (quint, *J* = 7.6 Hz, 2H), 1.39 (sext, *J* = 7.4 Hz, 2H), 0.93 (t, *J* = 7.4 Hz); ^{13}C NMR (100 MHz, $CDCl_3$) δ 148.28, 143.10, 134.84, 129.70, 128.72,

127.30, 120.96, 108.85, 31.43, 25.28, 22.29, 13.78; HRMS (ESI) [M + H^+] calcd for $C_{16}H_{19}N_3$, 228.1501; found, 220.1501.

1-[1-(Naphthalen-2-yl)vinyl]-4-phenyl-1*H*-1,2,3-triazole (12c)

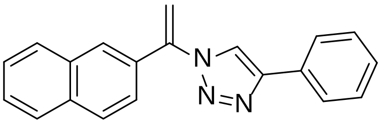

Compound **12c** was prepared in 75% yield as a yellow oil; ^{1}H NMR (400 MHz, $CDCl_3$) δ 7.92–7.81 (m, 7H), 7.57–7.53 (m, 2H), 7.48 (dd, *J* = 8.6, 1.8 Hz, 1H), 7.46–7.40 (m, 2H), 7.38–7.32 (m, 1H), 5.95 (d, *J* = 0.9 Hz, 1H), 5.70 (d, *J* = 0.9 Hz, 1H); ^{13}C NMR (100 MHz, $CDCl_3$) δ 148.0, 143.4, 134.1, 133.3, 132.2, 130.6, 129.2, 129.1, 128.8, 128.7, 128.1, 127.6, 127.5, 127.3, 126.2, 124.7, 120.3, 110.3; HRMS (ESI) [M + H^+] calcd for $C_{20}H_{16}N_3^+$, 298.1344; found, 298.1344.

4-(4-Bromophenyl)-1-(1-phenylvinyl)-1*H*-1,2,3-triazole (12d)

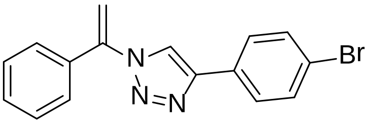

Compound **12d** was prepared in 55% yield as a yellow oil; ^{1}H NMR (400 MHz, $CDCl_3$) δ 7.79 (s, 1H), 7.72 (d, *J* = 8.6 Hz, 2H), 7.55 (d, *J* = 8.6 Hz, 2H), 7.49–7.36 (m, 5H), 5.87 (d, *J* = 1.1 Hz, 1H), 5.56 (d, *J* = 1.1 Hz, 1H); ^{13}C NMR (100 MHz, $CDCl_3$) δ 146.58, 142.89, 134.50, 132.01, 129.97, 129.17, 128.91, 127.34, 127.30, 122.25, 119.86, 109.56; HRMS (ESI) [M + H^+] calcd for $C_{16}H_{13}BrN_3^+$, 326.0293; found, 326.0293.

4-Benzyl-1-(1-phenylvinyl)-1*H*-1,2,3-triazole (12e)

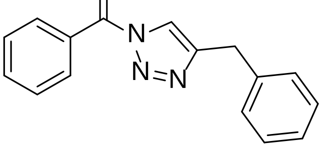

Compound **12e** was prepared in 63% yield as a colorless oil; ^{1}H NMR (400 MHz, $CDCl_3$) δ 7.35–7.43 (m, 3H), 7.20–7.33 (m, 8H), 5.74 (s, 1H), 5.48 (s, 1H), 5.14 (s, 2H); ^{13}C NMR (100 MHz, $CDCl_3$) δ 143.3, 138.9, 134.8, 129.9, 128.9, 128.8, 128.8, 127.4, 126.7, 109.3, 33.3; HRMS (ESI) [M + H^+] calcd for $C_{17}H_{16}N_3^+$, 262.1344; found, 262.1344.

4-(3-Chloropropyl)-1-(1-phenylvinyl)-1*H*-1,2,3-triazole (12f)

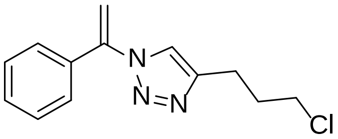

Compound **12f** was prepared in 48% yield as a yellow oil; ^{1}H NMR (400 MHz, $CDCl_3$) δ 7.36–7.45 (m, 4H), 7.29–7.35 (m, 2H), 5.77 (s, 1H), 5.49 (s, 1H), 3.6 (t, *J* = 6.3 Hz, 2H), 2.92 (t, *J* = 7.5 Hz, 2H), 2.20 (quin, *J* = 7.0 Hz, 2H); ^{13}C NMR (100 MHz, $CDCl_3$) δ 146.4, 143.2, 134.8, 130.0, 128.9, 127.5, 121.7, 109.2, 44.3, 31.9, 22.8; HRMS (ESI) [M + H^+] calcd for $C_{13}H_{15}ClN_3^+$, 248.0955; found, 248.0955.

4-[1-(1-Phenylvinyl)-1*H*-1,2,3-triazol-4-yl]butan-1-ol (12g)

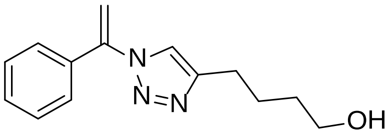

Compound **12g** was prepared in 41% yield as a yellow oil; ^{1}H NMR (400 MHz, $CDCl_3$) δ 7.28–7.43 (m, 6H), 5.75 (s, 1H), 5.47 (s, 1H), 3.67 (t, *J* = 6.3 Hz, 2H), 2.78 (t, *J* = 7.5 Hz, 2H), 1.76–1.82 (m, 2H), 1.64 (dt, *J* = 14.8, 6.4 Hz, 2H); ^{13}C NMR (100 MHz, $CDCl_3$) δ 148.6, 143.2, 132.9, 130.9, 128,9, 127.4, 121.3, 109.1, 62.4, 32.2, 25.6, 25.3 ppm; HRMS (ESI) [M + H^+] calcd for $C_{14}H_{18}N_3O^+$, 244.1450; found, 244.1454.

4-Butyl-1-[1-(naphthalen-2-yl)vinyl]-1*H*-1,2,3-triazole (12h)

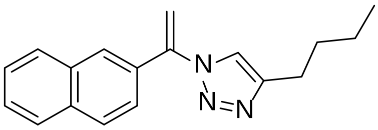

Compound **12h** was prepared in 53% yield as a yellow oil; ^{1}H NMR (400 MHz, $CDCl_3$) δ 7.88–7.81 (m, 3H), 7.78 (s, 1H), 7.55–7.52 (m, 2H), 7.42 (dd, *J* = 8.5, 2.0 Hz, 1H), 7.35 (s, 1H), 5.85 (s, 1H), 5.62 (s, 1H), 2.78 (t, *J* = 7.7 Hz, 2H), 1.69 (quint, *J* = 7.7 2H), 1.41 (sext, *J* = 7.4 Hz, 2H), 0.94 (t, *J* = 7.3 Hz, 3H); ^{13}C NMR (100 MHz, $CDCl_3$) δ 145.7, 143.2, 133.7, 132.9, 132.2, 128.6, 128.4, 127.7, 127.1, 127.1, 126.8, 124.4, 121.1, 109.4, 31.4, 25.3, 22.3, 13.8; HRMS (ESI) [M + H^+] calcd for $C_{18}H_{20}N_3^+$, 278.1657; found, 278.1657.

4-Benzyl-1-[1-(naphthalen-2-yl)vinyl]-1*H*-1,2,3-triazole (12i)

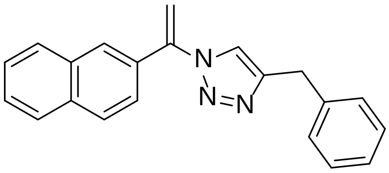

Compound **12i** was prepared in 59% yield as a colorless oil; ^{1}H NMR (400 MHz, $CDCl_3$) δ 7.87–7.79 (m, 3H), 7.76 (s, 1H), 7.56–7.49 (m, 2H), 7.39 (dd, *J* = 8.6, 1.8 Hz, 1H), 7.32–7.28 (m, 5H), 7.25–7.19 (m, 1H), 5.82 (d, *J* = 0.8 Hz, 1H), 5.61 (d, *J* = 0.8 Hz, 1H), 4.16 (s, 2H); ^{13}C NMR (100 MHz, $CDCl_3$) δ 147.9, 143.5, 139.1, 134.0, 133.3, 132.3, 129.0, 128.9, 128.7, 128.0, 127.5, 127.4, 127.0, 126.8, 126.5, 124.2, 121.9, 109.6, 32.2; HRMS (ESI) [M + H$^+$] calcd for $C_{21}H_{18}N_3^+$, 312.3877; found, 312.3878.

4-(4-Bromophenyl)-1-[1-(naphthalen-2-yl)vinyl]-1*H*-1,2,3-triazole (12j)

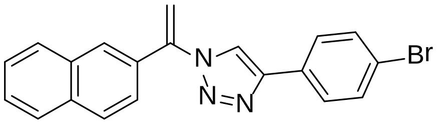

Compound **12j** was prepared in 47% yield as a colorless solid; mp 171 °C; ^{1}H NMR (400 MHz, $CDCl_3$) δ 7.91–7.82 (m, 5H), 7.73 (d, *J* = 8.6 Hz, 2H), 7.57–7.52 (m, 4H), 7.46 (dd, *J* = 8.6, 1.9 Hz, 1H), 5.94 (d, *J* = 1.1 Hz, 1H), 5.69 (d, *J* = 1.1 Hz, 1H); ^{13}C NMR (100 MHz, $CDCl_3$) δ 146.6, 142.9, 133.8, 133.0, 132, 131.7, 129.2, 128.8, 128.4, 127.7, 127.3, 127.3, 127.1, 127.0, 124.3, 122.3, 120.0, 110.0; HRMS (ESI) [M + H$^+$] calcd for $C_{20}H_{15}BrN_3^+$, 376.0449; found, 376.0451.

4-(4-Bromophenyl)-1-[1-(4-methoxyphenyl)vinyl]-1*H*-1,2,3-triazole (12k)

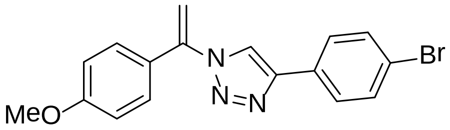

Compound **12k** was prepared in 51% yield as a yellow oil; ^{1}H NMR (400 MHz, $CDCl_3$) δ 7.80 (s, 1H), 7.72 (d, *J* = 8.5 Hz, 2H), 7.54 (d, *J* = 8.5 Hz, 2H), 7.29 (d, *J* = 8.8 Hz, 2H), 6.93 (d, *J* = 8.8 Hz, 2H), 5.74 (d, *J* = 1.0 Hz, 1H), 5.46 (d, *J* = 1.0 Hz, 1H); ^{13}C NMR (100 MHz, $CDCl_3$) δ 161.2, 146.8, 142.9, 132.3, 129.6, 129.1, 127.6, 127.2, 122.5, 120.3, 114.6, 108.3, 55.8; HRMS (ESI) [M + H$^+$] calcd for $C_{17}H_{15}BrN_3O^+$, 356.0398; found, 356.0395.

(*E*)-3-(4-Benzyl-1*H*-1,2,3-triazol-1-yl)-3-phenylprop-2-en-1-ol (12l)

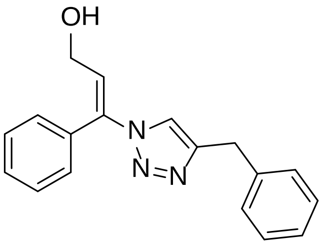

Compound **12l** was prepared in 39% yield as a yellow oil; ^{1}H NMR (400 MHz, $CDCl_3$) δ 7.47–7.40 (m, 3H), 7.32–7.20 (m, 8H), 6.62 (t, *J* = 7.2 Hz, 1H), 4.30 (d, *J* = 7.2 Hz, 2H), 4.10 (s, 2H); ^{13}C NMR (100 MHz, $CDCl_3$) δ 147.7, 139.0, 137.9, 132.8, 130.2, 129.6, 129.1, 129.0, 128.9, 126.9, 123.5, 121.8, 59.2, 32.4; HRMS (ESI) [$M + H^+$] calcd for $C_{18}H_{18}N_3O^+$, 292.1450; found, 292.1453.

References

1. Terent'ev, A. O.; Krylov, I. B.; Kokorekin, V. A.; Nikishin, G. I. *Synth. Commun.* **2008,** *38*, 3797–3809. doi:10.1080/00397910802226572
2. Padwa, A.; Dharan, M.; Smolanoff, J.; Wetmore, S. I. *J. Am. Chem. Soc.* **1973,** *95*, 1945–1954. doi:10.1021/ja00787a039
3. Zhao, W.; Carreira, E. M. *Chem.–Eur. J.,* **2006,** *12*, 7254–7263. doi:10.1002/chem.200600527
4. Shi, F.; Waldo, J. P.; Chen, Y.; Larock, R. C. *Org. Lett.* **2008,** *10*, 2409–2412. doi:10.1021/ol800675u
5. Wang, Y.-F.; Toh, K. K.; Chiba, S.; Narasaka, K. *Org. Lett.* **2008,** *10*, 5019–5022. doi:10.1021/ol802120u
6. Jordan, D. *J. Org. Chem.* **1989,** *54*, 3584–3587. doi:10.1021/jo00276a017
7. Duan, H.; Yan, W.; Sengupta, S.; Shi, X. *Bioorg. Med. Chem. Lett.* **2009**, *19*, 3899–3902. doi:10.1016/j.bmcl.2009.03.096
